# Advances in Targeted Toxin Therapy for Malignant Gliomas: A Narrative Review

**DOI:** 10.3390/toxins18040169

**Published:** 2026-03-31

**Authors:** Hanish Polavarapu, Walter A. Hall

**Affiliations:** Department of Neurosurgery, State University of New York Upstate Medical University, Syracuse, NY 13210, USA

**Keywords:** glioma, targeted toxin therapy, convection-enhanced delivery, receptor-targeted therapy, immunotoxin

## Abstract

Malignant gliomas remain highly treatment-resistant brain tumors despite surgery and adjuvant therapies. Targeted toxin therapies represent a unique strategy that exploits receptor-mediated cellular internalization to deliver cytotoxic components that result in the irreversible inhibition of protein synthesis independent of DNA damage or cell-cycle status. Advances in molecular profiling, toxin engineering, and delivery development have refined components targeting IL4Rα, IL13Rα2, EGFR/EGFRvIII, uPAR, and the transferrin receptor. Early clinical studies demonstrated biological activity, acceptable safety, and durable responses in subsets of patients, validating the fundamental mechanism of this approach. However, late-phase trials failed to demonstrate a population-level survival benefit, largely due to variability in delivery, receptor heterogeneity, and limitations in trial design rather than insufficient cytotoxic potency. Recent progress has focused on multiple receptor-targeting and delivery systems capable of achieving reliable intratumoral distribution. MRI-guided convection-enhanced delivery, vector-mediated toxin expression, and blood–brain barrier penetrant nanocarriers now enable more precise tumor targeting. Emerging evidence also reveals that toxin-mediated cytotoxicity can enhance antitumor immune responses, supporting their integration with immunotherapy. These advances position targeted toxins as precision cytotoxic compounds whose success depends on coordinated molecular targeting, delivery optimization, and biologically stratified patient selection, establishing a translational pathway for future glioma therapy.

## 1. Introduction

Malignant gliomas are aggressive primary central nervous system (CNS) tumors that include WHO grade 3 anaplastic astrocytoma and oligodendroglioma, and grade 4 astrocytoma or glioblastoma multiforme (GBM) [1]. These tumors originate from glial cells and are recognized for their infiltrative nature, genetic instability, and the ability to recur despite treatment [1]. Treatment of these tumors through surgery remains challenging due to their infiltration well beyond the radiographically visible tumor margin, resulting in a recurrence rate of over 90 percent in the case of GBM, despite obtaining a clinically apparent gross total resection [1]. The WHO classification of CNS tumors released in 2021 further refines this group of tumors, separating them based on molecular markers that include isocitrate dehydrogenase (IDH1/2) mutation status and 1p/19q codeletion that define two broad categories: IDH-mutant astrocytoma/oligodendrogliomas and IDH-wildtype GBMs [1,2]. While the prognosis may be impacted by tumor genotype, the overall survival for all malignant gliomas remains approximately 14 to 16 months for IDH-wildtype GBM and 4 to 7 years for IDH-mutant gliomas [1,3].

Current management of malignant gliomas involves a multidisciplinary approach that combines surgical resection and fractionated radiation therapy with temozolomide being administered concurrently as a radiosensitizer, followed by adjuvant temozolomide chemotherapy using a standard dosing regimen [4]. An optimal surgical resection and radiotherapy with temozolomide (TMZ) chemotherapy at a reduced dose was formalized in the Stupp protocol nearly two decades ago [4]. Although TMZ has been shown to modestly prolong survival, its overall effect is dependent on suppressing O^6^-methylguanine-DNA methyltransferase (MGMT), a DNA repair enzyme functional in over half of all GBM patients, which provides resistance to the alkylating mechanism of action of temozolomide [5]. These limitations have led to renewed interest in new treatment modalities for GBM that include targeted toxin (TT) therapy.

Early experimental efforts introduced the possibility of selectively delivering cytotoxic enzymes to tumor cells by exploiting specific cell surface receptors. These initial constructs, often termed immunotoxins, typically consisted of a carrier ligand conjugated to bacterial toxins such as the diphtheria toxin (DT) or Pseudomonas aeruginosa exotoxin A (PE). By exploiting tumor-expressed receptors, TTs obtain receptor-mediated internalization followed by the intracellular release of a catalytic toxin that ultimately results in the irreversible inhibition of protein synthesis, cellular apoptosis, or disruption of intracellular trafficking [6]. Although these initial agents demonstrated a potent anti-tumor effect in vitro and in small animal models, clinical translation was hindered by immunogenicity-limited tumor penetration, off-target toxicity, and inconsistent distribution [6].

Contemporary agents developed over the past decade have undergone fundamental structural and mechanistic changes. Receptor-directed fusion toxins, also known as receptor-directed TT, utilize binding ligands with high specificity [6]. These include cytokine domains or engineered protein scaffolds that are coupled with genetically modified toxin fragments. To further enhance safety, the native toxin receptor binding domain can be removed or replaced, preventing nonspecific uptake and ensuring that internalization primarily occurs through the engineered ligand–receptor interaction [7]. Advances in drug delivery and identification of over-expressed and spatially accessible glioma-associated receptors have strengthened the feasibility of directed TT [8,9].

A variety of cytotoxic protein scaffolds, including bacterial toxins such as DT and PE, as well as plant-derived ribosome-inactivating proteins and venom-derived peptides, have been developed and studied for glioma-directed TT. Other cytotoxic mechanisms explored for targeted delivery to malignant gliomas include highly potent bacterial AB5 toxins, engineered cytotoxic proteins, and venom-derived peptides [10,11]. While many of these agents are still experimental, they demonstrate the diverse cytotoxic mechanisms adapted for targeted delivery to malignant gliomas, highlighting the interest in non-DNA-dependent treatments for these tumors.

This review focuses on the advances in direct receptor-mediated TTs for malignant gliomas between 2015 and 2025, emphasizing the targeted receptors and summarizing the mechanistic, translational, and clinical progress of TTs in the context of emerging treatments for high-grade gliomas.

## 2. Overview and Historical Evolution of Toxin-Based Therapy in Gliomas

### 2.1. Fundamental Mechanisms of Protein Toxins

The therapeutic application of toxins in oncology derives from their exceptional catalytic potency and distinct mechanism of action. Unlike conventional chemotherapeutic agents, which typically require repeated exposure and depend on cell cycle dynamics, protein toxins act enzymatically within the cytosol and can induce cell death following delivery of only a few molecules per cell [12]. This property makes them particularly attractive for targeting heterogeneous and proliferating tumor populations such as malignant gliomas. Bacterial toxins, including DT and *PE*, mediate cytotoxicity through ADP-ribosylation of elongation factor-2 (EF-2), thereby irreversibly inhibiting protein synthesis [13,14,15]. In contrast, plant-derived ribosome-inactivating proteins (RIPs), such as ricin and saporin, exert their effects through N-glycosidase activity that depurinates 28S ribosomal RNA, resulting in permanent disruption of ribosomal function [16,17]. These mechanisms are independent of DNA damage and thus circumvent resistance pathways associated with conventional therapies.

**Figure 1:**
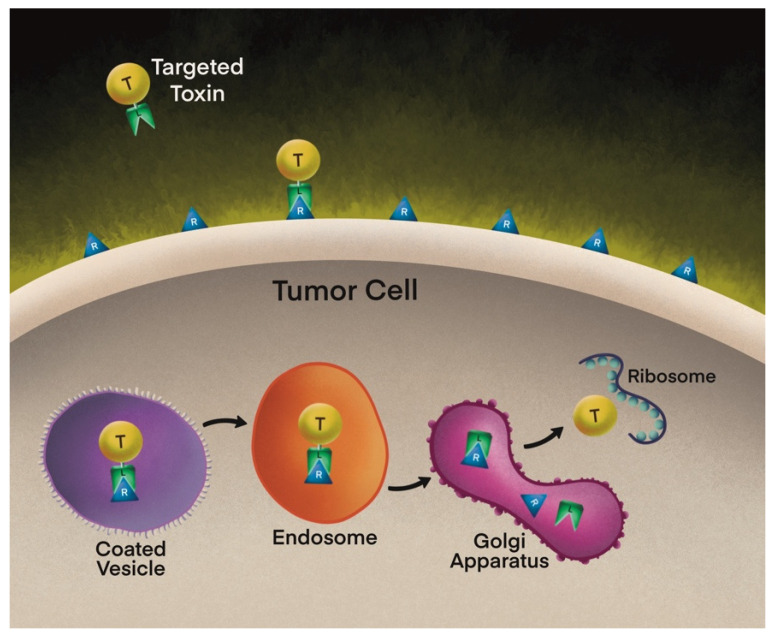
Targeted toxin mechanism of action. The targeted toxin comprises a carrier ligand conjugated to a mutated bacterial toxin. The carrier ligand recognizes a malignant cell-surface receptor. After binding to the receptor, the targeted toxin enters the cell in a coated vesicle, which then internalizes the construct in an intracellular endosome. The endosome migrates through the cytosol to the Golgi apparatus, where the toxin separates from the ligand. The toxin then localizes to the endoplasmic reticulum, where the ribosomes are located, and binds to elongation factor-2, thereby preventing the addition of amino acids to the growing polypeptide chain, resulting in cell death by inhibiting protein synthesis. T = toxin, L = carrier ligand, R = cell surface receptor.

Effective intoxication requires a coordinated sequence of events: (1) binding to a cell surface receptor, (2) internalization via endocytosis, (3) intracellular trafficking, and (4) translocation of the catalytic domain into the cytosol (Figure 1). Importantly, these intracellular pathways are toxin-specific. DT undergoes receptor-mediated endocytosis followed by pH-dependent translocation from acidified endosomes into the cytosol [13,18]. PE-based toxins, in contrast, traffic through retrograde pathways involving the Golgi apparatus and endoplasmic reticulum (ER), where sequences such as KDEL facilitate ER retention and subsequent cytosolic entry [19]. RIP toxins, including saporin, appear to enter the cytosol through Golgi-independent mechanisms that remain incompletely characterized [20,21]. These mechanistic differences have profound implications for toxin design, intracellular efficiency, and therapeutic specificity.

### 2.2. Early Development of Immunotoxins

The concept of redirecting toxin activity toward cancer cells emerged in the late 20th century with the development of immunotoxins that were composed of a targeting moiety linked to a cytotoxic protein. Initial approaches relied on chemical conjugation of monoclonal antibodies to toxin A chains, thereby enabling selective binding to tumor-associated antigens [22,23]. These early constructs demonstrated that selective cytotoxicity could be achieved in vitro and in preclinical models, establishing proof-of-principle for receptor-directed toxin therapy. In addition to antibody-based targeting, ligand-directed approaches were developed using growth factors such as epidermal growth factor (EGF) and interleukin-2 (IL-2), which bind receptors overexpressed in malignant cells [24,25]. These strategies expanded the repertoire of potential targets and highlighted the importance of receptor density and internalization kinetics in determining therapeutic efficacy. Hematologic malignancies, accessible to intravenously administered agents and often occurring with attenuated humoral immunity, provided early proof-of-concept [26]. However, early immunotoxins faced significant translational challenges. Their large molecular size limited penetration into solid tumors, particularly in the context of elevated interstitial pressure and heterogeneous vascular permeability [27]. Furthermore, inefficient intracellular trafficking and rapid immune-mediated neutralization reduced therapeutic durability. These limitations underscored the need for advances in both molecular design and delivery methodology. Key seminal studies that established the biological and translational foundation of toxin-based therapy in gliomas are summarized in Table 1.

### 2.3. Engineering Advances in Toxin Design

A breakthrough in the evolution of TTs was the ability to decouple toxin binding from cytotoxic function. Native toxins contain intrinsic receptor-binding domains that mediate nonspecific cellular uptake, resulting in substantial off-target toxicity [7,44]. Removal or mutation of these domains eliminates native cell binding while preserving the catalytic and translocation functions required for cytotoxic activity [7,44]. This modification allows the targeting ligand to fully dictate cellular specificity through receptor-mediated internalization. Structure–function analyses revealed that distinct domains within toxin molecules mediate receptor binding, translocation, and catalytic activity [45,46]. This enabled targeted modification or deletion of native binding domains while preserving intracellular cytotoxicity. One of the most influential developments was the generation of diphtheria toxin mutants such as CRM107, in which point mutations significantly reduced native receptor binding while maintaining enzymatic activity [47]. When coupled to tumor-specific ligands, these constructs demonstrated markedly improved selectivity and reduced systemic toxicity. Significant tumor regression was observed in majority of patients that were evaluated, with minimal systemic toxicity, demonstrating both the feasibility and potential efficacy of this approach [47]. Similar strategies were applied to PE, where truncated variants lacking the binding domain (e.g., PE40, PE38) were engineered for use in recombinant fusion toxins [48]. The advent of recombinant DNA technology further advanced the field by enabling the production of fusion toxins in which targeting ligands are genetically linked to toxin domains. Compared to chemically conjugated immunotoxins, these recombinant constructs offer improved structural uniformity, stability, and scalability [48]. Importantly, they also allow for precise modification of toxin sequences to optimize intracellular trafficking and reduce immunogenic epitopes.

In addition, advances in tumor biology reshaped toxin-based therapy. Studies have demonstrated that high-grade gliomas overexpress cell surface receptors that are virtually absent in normal brain tissue [49,50]. This finding led to the development of second-generation fusion toxins in which PE or DT binding domains were replaced with cytokine ligands. These engineered ligands enabled selective internalization into glioma cells while minimizing off-target toxicity, with murine models experiencing tumor regression with the sparing of healthy brain tissue [10].

### 2.4. Delivery Challenges and the Emergence of Convection-Enhanced Delivery

Despite advances in molecular engineering, effective delivery of TTs to brain tumors remained a critical barrier. The blood–brain barrier (BBB) restricts systemic delivery of large molecules, while the dense extracellular matrix and elevated interstitial pressure within tumors limit diffusion-based distribution [27]. Convection-enhanced delivery (CED) was developed to overcome these limitations by using continuous positive-pressure infusion to generate bulk flow through the brain interstitium. This approach enabled uniform distribution of macromolecules over large tissue volumes, independent of diffusion gradients [27]. Preclinical studies demonstrated that CED could achieve distribution volumes several-fold greater than infusion volumes and allow penetration into both white and gray matter [28]. CED represented a paradigm shift in intracranial drug delivery and has become a cornerstone of TT therapy in gliomas. By enabling localized, high-concentration delivery while minimizing systemic exposure, this technique addressed one of the primary limitations of earlier therapeutic approaches.

## 3. Mechanistic Advances in Glioma Biology Shaping Modern Toxin Design

Over the past decade, genomic, transcriptomic, and spatial profiling have refined the understanding of glioma heterogeneity, demonstrating that canonical toxin targets are not uniformly expressed but instead localize to distinct cellular and microenvironmental niches [51,52]. For example, IL13Rα2 and uPAR are enriched within invasive and mesenchymal phenotypes, whereas EGFR and EGFRvIII cluster within proliferative tumor cores, and uPAR localizes to the leading edge of tumor infiltration [53,54,55,56,57]. This spatial and phenotypic heterogeneity challenges earlier assumptions of homogeneous receptor expression and supports the development of fusion toxins tailored to tumor architecture rather than single-target paradigms.

Building on previously described foundational trafficking mechanisms, TTs utilize distinct intracellular pathways depending on the toxin scaffold, with these differences influencing therapeutic efficiency. Following receptor-mediated endocytosis, PE-based constructs undergo retrograde transport from endosomes to the Golgi apparatus and subsequently to the ER, where translocation into the cytosol occurs [19]. This process has been exploited through incorporation of the C-terminal KDEL (Lys-Asp-Glu-Leu) sequence, which enhances ER retention via KDEL receptors and increases the likelihood of cytosolic delivery of the catalytic domain [58,59]. In contrast, DT-based constructs translocate directly from acidified endosomes into the cytosol through pH-dependent conformational changes in the translocation domain, bypassing Golgi-mediated trafficking [13,18]. Plant-derived RIPs, such as saporin, appear to enter the cytosol through Golgi-independent mechanisms and are not thought to require classical ER retrieval pathways, although these processes remain incompletely defined [20,21]. Importantly, endosomal escape represents a critical rate-limiting step across toxin classes, as inefficient translocation from endosomal compartments can significantly reduce cytosolic delivery despite adequate receptor binding [60,61]. Recognition of these mechanistic differences has informed toxin-specific optimization strategies rather than a uniform design paradigm.

Protease-activated protoxin strategies introduce an additional layer of tumor specificity by restricting toxin activation to proteolytically active tumor microenvironments. In these constructs, the catalytic domain or translocation interface is sterically masked by inhibitory peptide sequences or linker domains that prevent activation until proteolytic cleavage occurs [62,63]. Tumor-associated proteases, including urokinase-type plasminogen activator (uPA) and matrix metalloproteinases (MMPs), cleave these engineered linkers to release the active toxin selectively within tumor tissue [62,63,64]. In gliomas, uPA activity is enriched in uPAR-expressing invasive niches, providing spatially restricted activation of protoxins. This strategy ensures that cytotoxic activity is confined to tumor regions, thereby minimizing off-target toxicity while preserving intracellular potency once activated [65,66].

Genomic and epigenomic analyses further support the concept of differential antigen susceptibility across glioma subtypes. IDH-mutant gliomas exhibit distinct metabolic and transcriptional programs that may reduce the expression of certain cell surface targets and facilitate immune evasion [67,68,69]. Similarly, MGMT promoter methylation and chromatin remodeling influence tumor biology and may indirectly modulate receptor expression and therapeutic responsiveness [70,71,72,73]. These findings underscore the importance of aligning target selection with tumor molecular subtype and dynamic tumor states.

Advances in tumor immunology have clarified mechanisms underlying the immunogenicity of toxin constructs and informed strategies to mitigate host immune responses. Native bacterial toxin domains in immunotoxins are well documented to be highly immunogenic, with defined B- and T-cell epitopes that drive neutralizing antibody formation and limit repeat dosing [74,75,76]. To address this, engineered toxin variants have been developed in which these dominant epitopes are selectively removed or modified through site-directed mutagenesis while preserving structural domains required for intracellular trafficking and catalytic activity [75,77]. Additional strategies include humanization of targeting ligands, reduction in protein aggregation, and optimization of linker regions to minimize immune recognition. Clinical experience with receptor-TTs has demonstrated that immunogenicity remains a significant limitation in systemic delivery, further motivating the use of localized intracranial administration [78]. In this context, intracranial delivery leverages the relative immune privilege of the central nervous system, where reduced antigen presentation and attenuated humoral responses allow for more sustained local activity of toxin constructs [79].

Collectively, these advances redefine efficacy in TT therapy as a function of intracellular routing efficiency, tumor-restricted activation, and host immune modulation rather than catalytic potency alone. Integration of these principles with the molecular and spatial architecture of gliomas has enabled the development of more precise and context-dependent therapeutic strategies, positioning TTs as a biologically informed platform for the treatment of high-grade gliomas.

## 4. Progress in Target-Specific Toxin Platforms (2015–2025)

The following sections highlight key receptor-directed toxin platforms that have progressed from early clinical validation to next-generation refinement over the past decade.

### 4.1. IL4R-Directed Toxins

The interleukin-4 receptor alpha (IL4Rα) emerged as a glioma-associated target in the 1990s when malignant astrocytomas and glioblastomas were shown to express higher receptor levels than surrounding normal brain tissue [30,80]. IL-4 signals through IL4Rα, forming a type I receptor complex with the common γ-chain in hematopoietic cells or a type II complex with IL-13Rα1 in non-hematopoietic tissues [81]. Importantly, IL4Rα undergoes receptor-mediated internalization following ligand binding, a property that is critical for efficient intracellular delivery of toxin constructs [82]. Although not tumor-specific, IL4Rα overexpression on glioma cells and tumor-associated myeloid populations enables targeting of both malignant cells and components of the immunosuppressive microenvironment that contribute to tumor progression [83].

Early IL4–toxin fusion constructs established proof of principle for receptor-directed cytotoxicity [49]. IL4-PE constructs replaced the native PE binding domain with circularly permuted IL-4 to improve ligand orientation and enhance receptor-mediated internalization, thereby facilitating intracellular delivery of the catalytic toxin domain. Following internalization, and consistent with PE-based constructs, IL4-PE undergoes retrograde trafficking through the Golgi apparatus and ER, where cytosolic translocation enables ADP-ribosylation of elongation factor-2 and inhibition of protein synthesis [59]. Preclinical studies demonstrated selective cytotoxicity across glioma models with relative sparing of adjacent normal brain parenchyma [30].

Clinical translation utilized CED in patients with recurrent malignant glioma. Phase I/II studies demonstrated that IL4-PE could be safely infused into the brain, achieving high local concentrations with minimal systemic exposure [31,36,38,84] (Table 2). Treatment-related adverse events were primarily infusion-related, including cerebral edema, elevated intracranial pressure, and transient neurologic deficits [38,85,86]. These effects were generally steroid-responsive and consistent with local tissue and volume effects, with negligible circulating toxin levels detected [85,86]. Radiographic evidence of necrosis in subsets of patients supported on-target biologic activity. However, survival outcomes were modest and heterogeneous, and no definitive maximum tolerated dose was established [85,86]. Limitations in intratumoral distribution, catheter positioning, receptor heterogeneity, and construct immunogenicity restricted consistent therapeutic exposure [87,88]. In addition, variability in intracellular trafficking efficiency and endosomal escape may have further contributed to heterogeneous treatment responses despite adequate receptor expression. Collectively, these findings implicated delivery, distribution, and intracellular processing as the principal barriers to efficacy.

Between 2015 and 2025, IL4R-directed therapies have advanced with the development of MDNA55 (bizaxofusp), a next-generation IL4R-targeted fusion toxin. MDNA55 links an engineered high-affinity IL-4 superkine to a truncated PE toxin, improving receptor engagement and intracellular delivery relative to earlier constructs [43,89,90]. The agent targets IL4Rα on both glioma cells and tumor-associated macrophages and microglia, enabling combined tumor cytotoxicity and microenvironmental modulation. MDNA55 is delivered via magnetic resonance imaging (MRI) guided CED using stereotactically placed catheters with real-time visualization of infusate distribution [43,90]. This approach represents an important advancement over earlier techniques by enabling direct confirmation of intratumoral coverage and reducing variability associated with catheter placement and infusion dynamics.

Clinical evaluation has progressed to a phase IIb trial in recurrent, unresectable glioblastoma. Results suggest improved survival and tumor control, including partial responses, stable disease, and modified Response Assessment in Neuro-Oncology (mRANO) defined pseudoprogression [43]. Median overall survival was reported as 10.2 months in the intent-to-treat population, 11.6 months in the per-protocol cohort, and approximately 15 months in a predefined high-dose subgroup, with 12-month survival approaching 55% in this group [43]. Tumor control rates exceeded 80% among optimally dosed patients [43]. Subsequent matched external control analyses supported survival advantage and registrational-stage therapeutic evaluation.

The toxicity profile of MDNA55 remains consistent with locally delivered biologic therapies. Adverse events are predominantly neurologic and infusion-related, including cerebral edema, seizures, and focal deficits, with relatively limited systemic toxicity [43]. Improvements in catheter design, infusion planning, and real-time monitoring have resulted in more consistent and predictable delivery profiles compared to first-generation IL4-PE studies. Ongoing development efforts focus on phase III evaluation, improved patient selection based on IL4Rα expression and spatial distribution, and rational combination strategies integrating IL4R-directed toxins with immunomodulatory therapies and approaches designed to overcome microenvironment-mediated resistance.

### 4.2. IL13Rα2-Directed Toxins

Interleukin-13 (IL-13) signals through two receptor systems with distinct functional pathways. Canonical IL-13 signaling occurs via the type II IL-4 receptor, formed by IL-13Rα1 in complex with IL-4Rα [91,92]. This heterodimer activates JAK1/Tyk2 and downstream STAT6-dependent transcription associated with type-2 immune responses, tissue remodeling, and fibrosis [91,92]. In contrast, IL13Rα2 is a high-affinity IL-13-binding receptor that is minimally expressed in normal tissues but frequently overexpressed in gliomas and other malignancies [30]. Although originally characterized as a decoy receptor with its short cytoplasmic domain and ligand sequestration function, subsequent studies demonstrated that IL13Rα2 can undergo receptor-mediated internalization and participate in non-canonical signaling pathways in cancer cells [93,94]. These include activation of AP-1, FAK, Src, and TGF-β-associated pathways, promoting tumor invasion, migration, and mesenchymal phenotypes [95]. In glioma, IL13Rα2 expression correlates with aggressive behavior and provides a relatively tumor-restricted entry point for TT delivery [95]. Importantly, its capacity for internalization enables efficient intracellular delivery of conjugated toxin constructs.

Unlike the broadly distributed IL-13/IL-4 heterodimeric receptor complex, IL13Rα2 exhibits a tumor-associated expression pattern and is strongly linked to aggressive biological behavior, including mesenchymal transition, invasive growth, and poor clinical outcomes [95,96]. Subsequent molecular characterization further clarified its distribution, demonstrating preferential expression in IDH-wildtype glioblastoma, TERT-mutant tumors, diffuse midline gliomas with H3K27M mutations, and glioma stem cell-like populations [97,98,99,100]. This convergence positioned IL13Rα2 as a compelling target for toxin-based therapy. Initial fusion constructs were created by linking IL-13 to truncated PE, such as IL13-PE38QQR (cintredekin besudotox) [36]. These constructs exhibited high-affinity binding to IL13Rα2, efficient receptor-mediated internalization, and potent cytotoxicity [101]. Following internalization, IL13-PE constructs undergo retrograde trafficking through the Golgi apparatus and ER, enabling cytosolic translocation of the catalytic domain and subsequent EF-2 ADP-ribosylation, consistent with other PE-based toxins [7]. Preclinical models consistently demonstrated regression of IL13Rα2-positive xenografts with sparing of receptor-negative tissues, supporting clinical translation [10,101,102].

Initial human studies utilized intratumoral CED to achieve localized drug distribution. Phase I and II trials demonstrated feasibility, acceptable safety, and biological activity, including radiographic necrosis and focal tumor regression in subsets of patients [36,101,103] (Table 3). Median survival in recurrent glioblastoma cohorts generally ranged from 10 to 12 months, with adverse events including thromboembolism, cerebral edema, and transient neurologic deficits [36]. However, therapeutic responses were inconsistent due to variability in catheter placement, catheter tract reflux, and limited diffusion of recombinant proteins [36]. These factors resulted in heterogeneous intratumoral distribution and inconsistent target engagement. These limitations were further highlighted in the PRECISE phase III trial comparing cintredekin besudotox with Gliadel wafers in recurrent GBM [39]. Median overall survival did not differ between treatment groups. Post hoc analyses suggested that delivery limitations were a major contributing factor, as a substantial proportion of catheters were suboptimally positioned and drug distribution was frequently insufficient to achieve therapeutic intratumoral concentrations [39]. These findings shifted focus away from large-scale trials of first-generation recombinant IL13-based toxins toward refinement of delivery strategies and construct design.

Between 2015 and 2025, development of IL13Rα2-directed toxins has focused on both molecular and delivery optimization. Molecular engineering efforts produced IL-13 mutants with increased affinity for IL13Rα2 and reduced binding to IL13Rα1/IL4R complexes, improving selectivity and limiting off-target uptake [105,106]. Dual-target constructs capable of binding both IL13Rα2 and EGFR were developed to address intratumoral heterogeneity, enhancing apoptotic signaling, and demonstrating potent cytotoxicity in stem cell-like tumor populations [98,106,107]. In parallel, delivery strategies expanded beyond direct protein infusion. Gene- and cell-based platforms, including viral vector-mediated expression systems and engineered cellular delivery approaches, have been investigated to enable sustained intraparenchymal toxin production and mitigate the short half-life and distribution limitations associated with recombinant proteins [40,108].

Clinical studies during this period have largely focused on locoregional and translational reassessment rather than late-phase trials. Combined IL13Rα2- and EphA2-directed toxin infusion studies confirmed feasibility and acceptable safety but unfortunately, demonstrated variable biological responses, reflecting persistent delivery constraints [103]. Another clinical series in pediatric and adult high-grade glioma populations similarly demonstrated feasibility with limited durability of response [104]. Across cohorts, effective biological activity correlated strongly with catheter placement accuracy, tissue permeability, receptor expression levels, and likely intracellular trafficking efficiency, emphasizing the importance of both spatial delivery and cellular processing in determining therapeutic outcomes [103,104].

An important translational development emerged from MRI-guided CED studies in dogs with spontaneous IL13Rα2- and EPHA2-positive gliomas [41]. These studies demonstrated reproducible intratumoral distribution, high target coverage, and substantial antitumor effects without dose-limiting neurotoxicity [41]. Objective decreases in tumor volume, including near-complete responses, were observed in a substantial proportion of treated animals [41]. These results support the concept that IL13Rα2-directed toxins possess strong intrinsic antitumor activity when delivery is spatially adequate, supporting the notion that prior human trial failures reflected technical and distributional constraints rather than mechanistic limitations.

Across studies from this period, consistent barriers to clinical success for IL13Rα2-directed toxin therapies have been identified, including suboptimal catheter placement, restricted volume of distribution due to reflux and tissue anisotropy, receptor heterogeneity, lack of receptor-based patient selection, and absence of real-time infusion monitoring [104]. Additionally, variability in intracellular trafficking and endosomal escape may further influence treatment response [106,109]. Collectively, these findings explain the disconnect between robust preclinical efficacy and modest clinical outcomes. IL13Rα2-directed toxins are therefore best conceptualized as delivery-dependent cytotoxic platforms whose efficacy is governed by spatial distribution, receptor density, and intracellular processing rather than intrinsic toxin potency alone.

### 4.3. EGFR-Directed Toxins

Recombinant constructs that bind wild-type EGFR (EGFRwt) and mutant EGFR variant III (EGFRvIII) to potent catalytic toxins, such as *PE* or DT, have been the focus of EGFR-directed toxin therapy for gliomas [55,110,111,112]. However, these toxins cannot be delivered systemically due to the risk of off-target toxicity and BBB exclusion [113]. Accordingly, development has relied on locoregional CED to achieve intratumoral distribution while minimizing systemic exposure.

EGFR is a transmembrane receptor composed of an extracellular ligand-binding domain, a transmembrane segment, and an intracellular tyrosine kinase domain [111]. Ligand binding induces receptor dimerization and rapid receptor-mediated internalization, a key property enabling efficient intracellular delivery of toxin constructs [114]. EGFRvIII arises from an in-frame deletion of exons 2–7, resulting in truncation of the extracellular domain and formation of a novel glycine residue at the fusion junction [112]. This structural alteration abolishes ligand binding and confers constitutive, ligand-independent low-level tyrosine kinase signaling activity [55,112]. EGFR amplification or overexpression occurs in approximately 50% of IDH-wildtype glioblastomas and is associated with aggressive tumor growth, resistance to therapeutic interventions, and a poor overall prognosis [115]. However, molecular profiling has demonstrated that EGFR expression is heterogeneous and dynamically regulated, with higher expression in proliferative tumor cores and reduced expression in invasive and mesenchymal regions [116]. Furthermore, EGFRvIII is now recognized as a subclonal and plastic alteration, with loss or downregulation observed under therapeutic pressure [117]. These insights have shifted therapeutic development toward dual-specific and multi-receptor constructs (capable of targeting more than one tumor-associated receptor) to address target-antigen heterogeneity.

Early constructs such as the EGFR-targeted PE fusion protein NB1-3001 and DT-based constructs (e.g., DT-EGF) demonstrated promising preclinical activity but limited clinical translation [110]. D2C7-IT is an EGFR-directed recombinant immunotoxin formed by fusing a single-chain variable fragment derived from the D2C7 antibody to a truncated PE toxin capable of recognizing both EGFRwt and EGFRvIII [110]. This architecture enables high-affinity receptor binding, efficient internalization, and subsequent inhibition of protein synthesis leading to rapid tumor cell death. Preclinical studies consistently demonstrated that D2C7-IT induces cytotoxicity in EGFRwt-, EGFRvIII-, and dual-expressing glioma cell lines [110]. This result led to robust tumor regression and survival prolongation in orthotopic xenograft models when delivered via intracerebral CED. Dual-ligand and bispecific constructs further enhanced tumor killing by engaging multiple receptor populations and activating convergent apoptotic pathways [118].

Early clinical experience with EGFR-directed toxins confirmed technical feasibility and biological activity but also revealed important translational constraints. Phase I and II intracerebral infusion studies demonstrated acceptable safety profiles, with toxicity dominated by local neurologic effects, including cerebral edema, seizures, and focal neurological deficits, and minimal systemic exposure [37] (Table 4). Radiographic responses were observed in subsets of patients; however, clinical benefit was limited by delivery variability and receptor heterogeneity. The most significant clinical advance with EGFR-directed toxins has been the translation of D2C7-IT to a phase I/II clinical trial in adult recurrent malignant glioma [119,120,121]. In this study, 43 patients were treated via intratumoral CED. Dose-limiting toxicities included seizures, pyramidal tract syndromes, confusion, significant cerebral edema, and dysphasia at higher dose levels [119]. A concentration of 6920 ng/mL in the infusate was selected for phase II evaluation [120]. Notably, a subset of patients demonstrated durable long-term responses, with survival extending beyond two to four years following a single infusion, suggesting that favorable alignment of receptor biology and delivery conditions can yield meaningful clinical benefit [119,120,121].

Between 2015 and 2025, progress in EGFR-directed toxin therapy has been driven primarily by biological refinement rather than expansion into late-phase trials. Development has emphasized dual targeting of EGFRwt and EGFRvIII, improved fusion protein engineering, and rational combination strategies informed by advances in tumor immunobiology. A growing body of translational work suggests that EGFR-directed toxins exert effects beyond direct cytotoxicity, functioning as immune-modulating agents within the tumor microenvironment [124]. These effects complement the direct mechanism of toxin-mediated protein synthesis inhibition. In immunocompetent murine glioma models, D2C7-IT prolonged survival in a T-cell-dependent manner, with depletion of CD4+ or CD8+ T cells resulting in reduced therapeutic efficacy [124]. Combination strategies pairing D2C7-IT with immune checkpoint blockade have produced complete tumor regression, systemic anti-tumor immunity, and protection against tumor rechallenge, indicating the induction of durable immune responses [121]. Additional studies combining D2C7-IT with CD40 stimulation demonstrated enhanced immune activation, including increased antigen presentation and sustained T-cell-mediated tumor control [121,124]. These effects are thought to arise from toxin-induced immunogenic cell death and enhanced antigen release within the tumor microenvironment.

Supporting evidence from other tumor models further highlights the broader applicability of this approach. In glioma, receptor-targeted proapoptotic strategies have been shown to sensitize tumor cells, including glioma stem cell-like populations, to death receptor mediated apoptosis, particularly through DR5/TRAIL signaling pathways, supporting the concept that TTs can augment apoptotic susceptibility beyond direct inhibition of protein synthesis [125,126]. In parallel, EGFR-directed toxin strategies in head and neck squamous cell carcinoma (HNSCC) models have demonstrated that bivalent EGF fusion toxin constructs achieve superior in vivo efficacy and reduced off-target toxicity compared with monovalent designs, including enhanced suppression of tumor growth and metastatic spread in EGFR-positive tumors [127]. Collectively, these findings redefine EGFR-directed toxins as dual-function agents that integrate targeted cytotoxicity with modulation of anti-tumor immune responses, rather than as standalone tumoricidal therapies.

### 4.4. uPAR-Directed Toxins

Urokinase-type plasminogen activator receptor (uPAR) is a key regulator of extracellular matrix remodeling, angiogenesis, and tumor–stromal interaction [128,129]. This functional role distinguishes it from receptors such as IL13Rα2, IL4R, and EGFR, which primarily serve as tumor-associated entry points for targeted therapies [130]. uPAR expression is low in normal brain and low-grade gliomas but markedly upregulated in anaplastic astrocytomas and GBM, where it correlates with mesenchymal differentiation, stem cell-like phenotypes, and adverse clinical outcomes [130,131]. Functionally, uPAR coordinates proteolysis through plasminogen activation, regulates integrin-mediated signaling, and promotes migratory and invasive behavior [132,133]. Its enrichment within invasive tumor margins and perivascular niches positions uPAR as a biologically relevant target for eliminating tumor cell populations that are less accessible to conventional therapies. Furthermore, expression on both glioma cells and tumor-associated endothelial cells enables a single uPAR-directed toxin to simultaneously target tumor viability, invasion, and angiogenesis across multiple compartments of the tumor microenvironment [132].

The fundamental uPAR-directed toxin construct is DTAT, a recombinant fusion protein that links the amino-terminal fragment (ATF) of urokinase-type plasminogen activator to the catalytic and translocation domains of diphtheria toxin (DT) [33,134,135]. Importantly, the amino-terminal fragment (ATF) domain alone is sufficient for high-affinity binding to uPAR, eliminating the need for full-length urokinase or co-receptor engagement and enabling more precise receptor-specific targeting [134]. This domain mediates receptor binding and internalization independent of additional co-receptors, allowing efficient delivery of conjugated toxins. Preclinical studies using ATF-based constructs, including saporin-linked chimeras, have demonstrated that ATF alone can achieve selective cytotoxicity in uPAR-expressing tumor cells without the need for RAP or other auxiliary binding components [136]. This design reflects a broader principle in TT engineering, in which minimal ligand-binding domains are used to direct cellular specificity while preserving the catalytic function of the toxin. Preclinical studies demonstrated selective killing of uPAR-positive glioblastoma cell lines and tumor-associated endothelial cells, confirming both tumor-selective and anti-angiogenic activity. In xenograft models, DTAT induced regression of uPAR-expressing tumors with preservation of normal tissue architecture and limited systemic toxicity at therapeutic doses, establishing biological credibility for dual-compartment targeting [135,137].

To address intratumoral heterogeneity and expand cellular targeting beyond single-receptor approaches, bispecific fusion toxins such as DTAT13 were developed by integrating IL-13 into the uPA-based DT backbone [137,138]. This construct enables simultaneous targeting of uPAR and IL13Rα2 while preserving independent binding affinities for both receptors, allowing parallel receptor engagement rather than competitive inhibition. Preclinical evaluation demonstrated high cytotoxic potency across multiple glioma models, sustained tumor regression in xenografts, and antitumor efficacy in intracranial models comparable to monospecific constructs, with reduced systemic toxicity [137]. These findings established DTAT13 as an early example of multi-receptor toxin design aimed at improving tumor coverage while limiting off-target effects.

Despite a strong biological rationale and reproducible preclinical efficacy, uPAR-directed TTs have not progressed to late-phase clinical trials. This likely reflects translational and strategic limitations rather than inherent shortcomings of the target itself. uPAR expression is spatially enriched within invasive and perivascular tumor compartments, making effective targeting highly dependent on precise spatial drug distribution, which early CED systems were unable to consistently achieve [128,139,140]. In addition, early clinical development prioritized receptors considered primarily tumor-restricted entry points, whereas the broader functional role of uPAR in invasion, angiogenesis, and stromal signaling introduced complexity in trial design and response assessment [128].

As of 2025, uPAR-directed toxins remain in the preclinical domain without completed human glioma trials. Nonetheless, their contribution has been significant in shaping modern toxin design strategies. These constructs introduced durable design principles that now support next-generation toxin therapies, including multi-receptor targeting, invasion-focused biology, microenvironmental modulation, and toxicity reduction through receptor-sharing architectures. Across contemporary glioma-toxin literature, DTAT and DTAT13 are now cited as fundamental constructs that shape current multi-receptor toxin engineering, a uPAR-targeted imaging environment, and hybrid therapeutic strategies.

### 4.5. Transferrin Receptor (TfR)

Malignant gliomas exhibit high metabolic activity, with rapidly proliferating tumor cells requiring increased iron uptake. This demand leads to a corresponding upregulation of transferrin receptor (TfR) expression, whereas normal brain tissue expresses comparatively low receptor levels [141]. Transferrin receptor-directed toxin therapy in glioma is represented by Tf-CRM107 (TransMID), one of the earliest clinically translated TT constructs. This conjugate links human transferrin to a mutated DT variant (CRM107) and is designed to exploit transferrin receptor-mediated internalization for selective tumor cell killing [34]. CRM107 lacks its native receptor-binding domain while preserving catalytic activity, eliminating nonspecific uptake and rendering cytotoxicity entirely dependent on transferrin receptor engagement [34]. This design exemplifies a core principle of TT engineering, in which truncated toxin domains are used to remove native binding while allowing the targeting ligand to dictate cellular specificity.

Clinical investigation of Tf-CRM107 provided early evidence that receptor-TTs could exert measurable antitumor effects in glioma when delivered directly into tumor tissue. In initial locoregional infusion studies, a subset of patients experienced striking radiographic responses, including marked reductions in enhancing tumor volume and occasional complete responses, all in the absence of clinically significant systemic toxicity [29,34] (Table 5). These findings demonstrated effective receptor engagement and intracellular toxin activity in vivo, validating the transferrin receptor as a functional entry pathway for catalytic cytotoxins within the central nervous system. Follow-up phase I and II studies extended these observations across larger cohorts, demonstrating reproducible biological activity and a safety profile dominated by localized neurologic effects rather than systemic toxicity, with a median survival of approximately 8–9 months in recurrent malignant glioma [34].

The subsequent failure of the multicenter phase III TransMID trial marked a pivotal point in the field. A phase III multicenter, randomized study in recurrent, nonresectable GBM was initiated as the pivotal TransMID trial, with patient dosing beginning in 2004, but the program was later withdrawn before enrollment could continue after the phase II experience raised safety concerns. Interim analyses showed a radiographic response rate of approximately 39% and a median overall survival of approximately 37 weeks, without a clear improvement over contemporaneous standards of care, leading to discontinuation of the study [34,142]. Rather than undermining the biological rationale for TfR targeting, post hoc analyses and technical reviews identified significant limitations in drug delivery [87,143]. Variability in catheter positioning, restricted parenchymal distribution, reflux into cerebrospinal fluid spaces, and lack of real-time confirmation of infusion resulted in heterogeneous intratumoral exposure [34]. These factors were further compounded by large tumor size, the presence of significant tumor necrosis, and heterogeneous tissue permeability, which further impeded uniform toxin dissemination. Neurologic adverse events were subsequently recognized to be largely related to infusion dynamics and local tissue response rather than intrinsic toxin toxicity [87]. Collectively, these findings suggest that delivery limitations, rather than intrinsic deficiencies in the mechanism of action, were a major contributor to the negative trial outcome. As with other DT-based constructs, variability in endosomal escape efficiency may also have influenced intracellular toxin delivery and treatment response.

Although clinical development of Tf-CRM107 was discontinued after the phase II experience, its influence on the field has been enduring. The program provided one of the earliest demonstrations that large receptor-targeted cytotoxins could be administered safely within the brain and could induce meaningful tumor regression when adequate parenchymal distribution was achieved. In doing so, Tf-CRM107 established key principles that continue to define locoregional biologic therapy in neuro-oncology, where target engagement alone is insufficient, and therapeutic success depends on controlled, spatially verified intratumoral exposure.

Between 2015 and 2025, TfR has increasingly been explored as a transport mediator across the BBB rather than solely as a direct entry receptor for toxin conjugates. Research has focused on transferrin-decorated nanoparticles, ferritin-based delivery systems, polymeric carriers, and dual-function imaging–therapy constructs designed to exploit receptor-mediated transcytosis and preferential tumor uptake [144,145,146,147]. These approaches shift the role of TfR from a direct toxin entry receptor to a transport mechanism that facilitates delivery of diverse therapeutics across the BBB, enabling systemic or nose-to-brain delivery strategies that do not rely on DT-based conjugates.

### 4.6. Miscellaneous and Emerging Toxin Modalities

In addition to receptor-directed bacterial immunotoxins, a heterogeneous group of alternative toxin platforms has been explored in gliomas, primarily in preclinical and early translational settings [148]. These approaches expand toxin-based therapy beyond classical fusion proteins toward engineered cytotoxic platforms that integrate alternative cell-death mechanisms, tumor-specific activation, and advanced delivery strategies [149]. Although many of these modalities remain investigational, they provide important insight into next-generation design principles for targeted cytotoxic therapy.

Plant-derived RIPs, including ricin A chain, saporin, and gelonin, were among the earliest alternative toxins evaluated in glioma models [150,151,152,153] (62–64). These agents irreversibly inactivate ribosomes through N-glycosidase activity, leading to inhibition of protein synthesis and apoptosis independent of cell cycle status, making them particularly suited for targeting quiescent and stem cell-like tumor populations [150,151,154]. Unlike bacterial toxins such as PE, RIPs do not require retrograde trafficking through the Golgi apparatus and are thought to enter the cytosol through Golgi-independent mechanisms, although these pathways remain incompletely defined [150]. Because many RIPs lack intrinsic cell-binding domains, they function as modular cytotoxic platforms in which the catalytic domain is independent of the targeting ligand and can be selectively directed using antibodies or receptor-specific ligands [150,151]. When conjugated to targets such as EGFR, IL13Rα2, tenascin, or the transferrin receptor, these constructs have demonstrated potent cytotoxicity in vitro and in xenograft models [150,152].

To reduce immunogenicity, human-derived cytotoxic proteins such as granzyme B and engineered ribonucleases have also been investigated as alternative toxins [155,156,157,158]. These proteins lack foreign bacterial epitopes, reducing the likelihood of neutralizing antibody formation and improving compatibility with repeated dosing strategies [157,158]. Granzyme-based fusion constructs aim to mimic cytotoxic lymphocyte function by triggering caspase-dependent apoptosis following internalization [159]. However, compared with bacterial toxins, these agents exhibit lower intrinsic catalytic potency and remain largely preclinical in glioma, reflecting the need for highly efficient targeting and intracellular delivery to achieve meaningful therapeutic effects [115,157,158].

A major conceptual advance in this category has been the development of protease-activated pro-toxins that remain inactive until selectively activated within the tumor microenvironment [160,161]. These constructs incorporate inhibitory peptide sequences or masking domains that prevent toxin activity until cleaved by tumor-associated proteases, including matrix metalloproteinases, urokinase-type plasminogen activator, legumain, and cathepsins [160,161,162]. By introducing tumor-specific cleavage sites, these systems provide an additional layer of selectivity beyond receptor targeting alone, restricting cytotoxic activity to proteolytically active tumor regions.

One of the most rapidly evolving areas within this category is nanocarrier-based toxin delivery, which aims to enable systemic administration while maintaining intracranial specificity. Liposomes, polymeric nanoparticles, ferritin-based cages, and inorganic particles have been engineered to carry toxins or toxin-mimetic agents, with surface ligands that facilitate receptor-mediated transcytosis across the BBB and preferential tumor uptake [146,147,163,164]. These systems enhance circulation time, protect toxin stability, and improve intratumoral accumulation, offering a potential solution to the delivery limitations associated with direct toxin infusion.

## 5. Delivery, Clinical Translation, and Future Direction

### 5.1. Delivery Science: A Critical Determinant of Efficacy

Across all receptor-directed toxin constructs for high-grade gliomas, delivery has increasingly been recognized as a determinant of therapeutic success. Clinical outcomes have repeatedly been constrained by the ability to achieve adequate, spatially controlled intratumoral exposure [10,109,165,166]. This is often independent of receptor selection, ligand engineering, or toxin potency. Over the past decade, it has become apparent that the efficacy of TTs depends as much on their delivery technique as on the biological validity of the target itself [109]. CED, vector-mediated toxin expression, and systemic nanocarrier vehicles have therefore evolved from supporting technologies into core biological variables that govern efficacy, toxicity, and clinical translatability [165,167].

CED was developed to overcome the limitations of diffusion-based transport and the impermeability of the BBB by using continuous positive-pressure infusion to generate bulk interstitial flow [28,166]. Unlike diffusion-limited transport, which depends on concentration gradients and is inefficient for large macromolecules, convection enables pressure-driven bulk flow that is largely independent of molecular size [168,169]. Mechanistically, CED enables macromolecules such as toxins, antibodies, and viral vectors to achieve volumes of distribution that exceed the infused volume, allowing therapeutic agents to traverse extracellular spaces, white-matter tracts, and infiltrative tumor margins that are otherwise pharmacologically inaccessible [168,169,170]. This principle provides the biological foundation for locoregional toxin therapy in the central nervous system.

In practice, however, CED has proven highly sensitive to physical and anatomic variables that directly determine efficacy [140,168]. Reflux and backflow along the infusion catheter, leakage into cerebrospinal fluid spaces, pooling within necrotic tumor cavities, and barriers formed by pial surfaces or white-matter anisotropy can drastically reduce effective parenchymal distribution [140]. As a result, catheter geometry, placement trajectory, and tissue architecture become dominant determinants of drug delivery [140]. Clinical series consistently demonstrate wide variability in effective drug distribution, explaining why many patients in toxin trials derived little benefit despite receiving adequate nominal doses. In this context, insufficient spatial coverage rather than insufficient pharmacologic potency has been a major contributor to therapeutic failure [109]. Even when adequate distribution is achieved, therapeutic efficacy remains dependent on efficient intracellular trafficking and cytosolic delivery of the toxin.

Delivery engineering has therefore become critical to modern CED. Reflux-resistant, recessed-step, multiport, and arborizing catheter designs now allow larger volumes of distribution at lower flow rates, improving coverage of irregular tumor geometries while minimizing infusion backflow [171,172]. Image-guided, patient-specific catheter planning combined with intra-procedural MRI or computed tomography (CT) imaging using contrast tracers allows for real-time visualization of infusate distribution, allowing for immediate adjustment of catheter number, trajectory, and flow parameters [173,174]. Pressure-modulated and multipoint infusion systems distribute flow across multiple microports, reducing local tissue damage while preserving convective transport. Together, these advances transform CED from a blind infusion technique into a quantifiable, image-verified delivery platform, fundamentally improving its reliability and interpretability in clinical trials.

Gene- and vector-based delivery methods provide an alternative strategy for overcoming the pharmacokinetic limitations of protein toxins. Rather than delivering the toxin by itself, viral vectors introduce genetic constructs that encode toxin components directly into tumor tissue, enabling sustained local expression [108,175]. Adenovirus and lentivirus-based systems have been engineered to express receptor-TT constructs under drug-inducible promoters, often combined with co-expression of competitive or blocking ligands to reduce off-target receptor engagement [175]. These systems confer several biologically meaningful advantages. Sustained in situ expression overcomes the short half-life of infused proteins. Bystander effects, in which toxin-expressing or vector-transduced cells induce death in adjacent non-transduced tumor cells through diffusion of cytotoxic molecules or propagation of apoptotic signaling, and spatial amplification effects, whereby localized toxin production expands the effective treatment field beyond the initial delivery site, help address patchy transduction and infiltrative tumor growth [176]. Regulation and safety can be enhanced through inducible promoters that allow external control of toxin expression, reducing neurotoxicity relative to constitutive expression systems [177].

Ligand-targeted nanocarriers designed for toxin or toxin-mimetic delivery integrate multiple functional stages within a single construct, including vascular transport, brain penetration, tumor localization, cellular internalization, and intracellular release [178]. Modern systems incorporate protease-cleavable or pH-sensitive linkers so that the active cytotoxic component is released preferentially within the tumor microenvironment, reducing systemic exposure while maintaining high local potency [179]. Additional engineering strategies include de-immunization of toxin domains, shielding approaches, and multi-ligand targeting structures that combine blood–brain barrier transport ligands with tumor-specific receptors on the same carrier, thereby enhancing circulation time, facilitating receptor-mediated transcytosis across the blood–brain barrier, and ensuring that the active cytotoxin is released within tumor cells and reaches the cytosol to exert its effect [180].

### 5.2. Clinical Translation: Interpretation of Human Studies (2015–2025)

In studies published between 2015 and 2025, clinical experience with receptor-directed toxin therapies for high-grade gliomas followed a consistent translational pattern. Early-phase trials generally demonstrated biological activity, technical feasibility, and acceptable safety, yet late-phase studies have often not demonstrated a significant population-level survival benefit [165]. Importantly, efficacy was not absent but restricted to biologically and technically favorable subsets, characterized by adequate receptor expression, optimal catheter placement, and sufficient intratumoral drug distribution, indicating incomplete integration of targeting, delivery, and patient selection rather than mechanistic failure [34,36,39]. This pattern is exemplified by trials such as the PRECISE study of cintredekin besudotox and the TransMID trial of Tf-CRM107, in which promising early-phase signals were not reproduced in larger, multicenter settings [34,36,39]. Collectively, these findings underscore that clinical outcomes are governed by the integration of biologic targeting, delivery precision, and patient selection rather than any single component alone.

Improvements in median overall survival were relatively modest across various recurrent GBM cohorts, typically falling within the range of 9 to 12 months. However, certain agents, such as MDNA55 and D2C7-IT, showed consistent durable clinical responses in subsets of patients, leading to extended survival beyond 2 to 4 years in select patients following a single locoregional infusion [43,170]. These results highlight the potential to achieve enduring treatment responses when factors such as receptor density, tumor biology, and delivery conditions align. Overall, at the cohort level, treatment responses remained heterogeneous, with radiographic tumor necrosis and focal tumor control being common and sustained disease control being difficult to achieve [34,43]. It became evident that clinical benefit was correlated with delivery quality and biological enrichment rather than the nominal dose, as catheter placement, sufficient volume of distribution, and receptor-enriched tumors consistently predicted better outcomes [170]. Furthermore, toxicity profiles were similar across various constructs, primarily driven by local delivery rather than by the toxin itself. Neurologic side effects such as edema, seizures, and transient focal deficits were predominant, with minimal systemic toxicity, confirming the safety profile of receptor-directed toxins when administered intracranially [34,43,165,170,174]. The clinical risk associated with these treatments was primarily influenced by infusion dynamics and tissue response rather than molecular toxicity. Even when adequate distribution is achieved, therapeutic efficacy remains dependent on efficient intracellular trafficking and cytosolic activity of the toxin. However, the translation of these findings was hindered by several recurring biological constraints.

The expression of receptors in high-grade gliomas varies across different spatial locations within the tumor. Receptors such as IL13Rα2, IL4R, EGFR/EGFRvIII, uPAR, and the transferrin receptor are confined to specific areas within the tumor [181]. Targeting a single receptor often leaves large tumor regions untreated, necessitating the use of multi-receptor type strategies or spatially stratified delivery. Immunogenicity can limit repeated or systemic dosing, making single-session or limited-exposure locoregional therapy more clinically feasible. Tumor architecture, including size and invasiveness, significantly impacts the distribution and efficacy of treatment [182]. Large, necrotic, and highly infiltrative tumors have poor drug distribution and reduced effectiveness, while smaller, anatomically favorable recurrences respond in a more consistent manner [182]. The lack of success in Phase III trials is most plausibly explained as a limitation at a systems level, rather than a failure of the underlying biology or safety profile. Early-phase studies benefit from technical optimization, biologic enrichment, and delivery control, although these conditions are challenging to maintain consistently. In multicenter trials, variability in catheter placement, lack of distribution verification, and broad eligibility criteria dilute effective drug exposure and obscure biological signals [140,168]. Conventional trial designs further compound this issue by combining device performance and biologic efficacy into a single endpoint, which can make it difficult to distinguish between delivery failure and therapeutic inefficacy.

Collectively, the clinical literature supports that toxin-based therapy remains valid, but it also emphasizes the need for improved system integration. The remaining challenge lies in aligning molecular targeting, delivery engineering, imaging verification, patient selection, and trial design into a unified therapeutic framework. Future progress in this field will depend less on new toxins and more on biologically stratified enrollment, delivery-verified trials, and integrated multimodal strategies. Within this framework, toxins are not standalone drugs but precision cytotoxic components within coordinated neuro-oncologic treatment systems.

### 5.3. Integration with the Modern Therapeutic Landscape

TTs play a unique role in the current treatment landscape for malignant gliomas. Rather than employing traditional cytotoxic approaches, they offer a fundamentally different therapeutic strategy based on receptor-mediated internalization and irreversible disruption of intracellular processes, primarily protein synthesis [6]. This method of cytotoxicity distinguishes toxin-based therapies from conventional methods such as alkylating chemotherapy, radiation, and tumor-treating fields, positioning them as biologically complementary rather than incremental alternatives [183]. This distinction is clinically significant because standard therapies preferentially affect rapidly proliferating tumor cells and are constrained by well-established resistance mechanisms, including MGMT-mediated DNA repair and cellular quiescence [184,185]. In contrast, receptor-directed toxins work regardless of cell-cycle status, allowing them to direct target quiescent, stem cell-like, mesenchymal, and therapy-resistant cells that often drive recurrence [185]. Therefore, toxin-based agents should not be viewed as a replacement for standard therapy, but as additional cytotoxic adjuvants that can address biological niches that may have escaped existing treatments.

Primary antitumor activity of TTs is not reliant on the activation of the immune system. However, the death of tumor cells caused by toxins does not go unrecognized by immune cells. Toxins work largely by inhibiting protein synthesis and inducing intracellular stress, which in turn leads to the release of antigens and innate immune signaling. As a result, conditions are created that enhance the presentation of antigens and the responsiveness of the immune system. Studies have consistently demonstrated that toxin-induced reduction in tumor cell populations makes gliomas more sensitive to other immunotherapies that work through death-receptor agonists or costimulation of other immune pathways [186,187]. This ability positions toxins as agents that may be capable of conditioning the immune system and modulating the tumor microenvironment to support subsequent immune therapies.

The contrast between toxin-based therapies and systemic receptor-mediated approaches is best exemplified by antibody–drug conjugates (ADCs). Mechanistically, ADCs and ligand–toxin fusion proteins share a common framework that is built on receptor recognition, internalization, and deployment of the toxic portion with targeted cytotoxicity [188]. Both strategies are reliant on receptor density, endocytic trafficking, and intracellular processing to kill tumor cells. However, the key difference lies in the method of delivery. ADCs rely on vascular perfusion and permeability of the BBB to reach the target tumor, using small-molecule cytotoxic components rather than bacterial or plant toxins [189]. In gliomas, this may prove to be challenging. Heterogeneous disruption of the BBB, limited delivery to infiltrative tumor margins, and reduced penetration into non-enhancing tumor regions would severely restrict the effective distribution of ADCs, although the molecular targets are biologically appropriate [190].

Depatuxizumab mafodotin (Depatux-M, ABT-414) is a notable clinical example in the field of oncology. This EFGR-targeted ADC showed activity in EGFR-amplified GBM, leading to radiographic responses and signs of clinical benefit in specific patients. The INTELLANCE-2 trial results indicated possible improvement in outcomes when Depatux-M was administered in combination with temozolomide in patients with recurrent disease [191]. However, the INTELLANCE-1 phase III trial did not demonstrate any survival benefit in newly diagnosed GBM despite molecular stratification [192]. Subsequent analyses revealed translational limitations such as BBB-restricted drug exposure, especially in infiltrative and non-enhancing tissue. Furthermore, receptor heterogeneity diluted effective receptor engagement across the tumor mass and off-target toxicity, such as corneal epithelial injury from physiologic EGFR expression, narrowed the therapeutic window [192]. Retrospective data suggested potential benefits in MGMT-methylated or low-volume disease burden, but these were insufficient to overcome systemic constraints [192]. It should be noted that the failure of Depatux-M does not discredit the concept of receptor targeting biologically but rather highlights the challenges of systemic toxin delivery in the context of the anatomic and vascular pathology and microenvironmental physiology of gliomas. This distinction explains why intracranial toxin strategies have persisted, while systemic toxin approaches have faced significant challenges. Local delivery, by bypassing the BBB and enabling high intratumoral concentrations, offers advantages over systemic approaches in targeting infiltrative and non-enhancing disease compartments, which are often difficult to access using systemic delivery.

### 5.4. Future Directions

The next phase of toxin-based therapy development in malignant gliomas will focus less on introducing new cytotoxic compounds and more on enhancing biological precision, delivery engineering, and clinical integration [40,193]. After many years of demonstrating the concept, the primary challenge now is not whether TTs can eliminate glioma cells, but how to deploy them with precise spatial accuracy, molecular specificity, and suitable patient selection to achieve lasting clinical benefits [40]. Future methods are anticipated to advance from targeting a single receptor to utilizing multi-receptor structures that better represent the diverse nature of tumors and receptor adaptability [106,121]. Dual- and tri-specific constructs are expected to expand cellular coverage across various parts of the tumor while reducing the toxin burden on each receptor, thus improving safety and efficacy [194]. These developments are closely linked to advancements in tumor mapping technologies, which now allow for specific characterization of target expression within the tumor, enabling the engineering of toxins tailored to individual patient’s receptor landscapes. Delivery science will continue to play a crucial role in determining the effectiveness of toxin-based therapies, with ongoing advancements such as image-guided catheter placement, pressure-modulated infusion, and implantable microinfusion systems that enhance delivery precision and control [154]. Vector-mediated compounds offer additional benefits by enabling prolonged local toxin expression, bystander killing, and spatial amplification of cytotoxic effects, thereby partially uncoupling efficacy from physical catheter limitations and protein half-life [40,108]. Systemic administration, including BBB-penetrant nanocarriers and exosome-based systems, are expected to complement localized delivery methods, serving as supportive tools for priming and conditioning rather than standalone cytotoxic strategies [156,194]. For successful clinical translation, there needs to be a shift from empiric enrollment to biologically stratified trial design, with core eligibility criteria based on receptor density, spatial localization, tumor volume, necrosis, and tissue architecture. Ongoing molecular engineering efforts aim to further enhance safety and intracellular efficiency by developing humanized targeting domains, de-immunized toxin cores, and optimized intracellular trafficking mechanisms, thereby expanding therapeutic windows and enabling repeat or combination dosing without increasing systemic toxicity [7]. The path to late-phase clinical success is becoming clearer, with delivery variability, biological heterogeneity, and trial design misalignment identified as primary factors contributing to prior failures. Future trials are expected to treat toxin constructs, delivery systems, and patient selection as an integrated therapeutic unit, with endpoints that incorporate distribution verification and target engagement alongside survival outcomes. Toxin-based therapies are likely to function best within integrated, multimodal treatment systems, complementing immunotherapy, cellular therapies, radiation, and molecularly targeted agents, and positioning themselves as rational and technically mature components of future high-grade glioma therapy.

## 6. Conclusions

Toxin-based therapies for malignant gliomas have a strong biological rationale. Decades of experimental and clinical research have demonstrated that cytotoxins directed at specific receptors can effectively kill tumors by mechanisms different from traditional DNA-damaging treatments. These cytotoxins can kill tumor cells regardless of their cell-cycle status and DNA repair pathways, making them effective against quiescent, stem cell-like, and therapy-resistant tumor populations. The early translation of these findings into clinical applications was hindered by delivery limitations, receptor variability, and system-level integration issues. However, advancements in molecular engineering, delivery techniques, and tumor biology have addressed many of these initial challenges. Modern TT therapy now incorporates high-affinity targeting ligands, de-immunized toxin cores, multi-receptor architectures, and precise delivery systems for controlled intraparenchymal distribution and real-time spatial verification. This transformation has turned toxin therapy from a conceptually powerful but fragile strategy into a biologically coherent and technically feasible therapeutic platform. The key lesson learned is that the success of toxin-based therapies depends on the precise matching of the toxin, receptor target, delivery modality, and patient selection to the molecular architecture, spatial organization, and microenvironmental context of each tumor. When these elements are aligned, durable responses become biologically plausible; however, when misaligned, even potent biologic agents will fail. As neuro-oncology shifts towards mechanism-driven care, toxin-based therapies are best viewed as precision cytotoxic modules within integrated therapeutic systems, rather than standalone drugs. Their future clinical impact will be determined by the effective integration of molecular targeting, delivery engineering, and clinical strategy into a single translational framework. Within this framework, receptor-directed toxins hold a rational and durable position as spatially precise, biologically orthogonal, and mechanistically complementary components of next-generation multimodal therapy for high-grade glioma.

## Figures and Tables

**Table 1:** Seminal convection-enhanced delivery and toxin-based glioma studies. Key historical papers that established convection-enhanced delivery, validated major glioma receptor targets, and defined the clinical translation of targeted toxin therapy in malignant brain tumors.

Year	Author	Article	Target/Platform	Contribution
1994	Bobo et al. [28]	Convection-enhanced delivery of macromolecules in the brain	CED methodology	Foundational CED paper establishing the bulk-flow, pressure-driven delivery principle for intraparenchymal CNS therapy.
1997	Laske et al. [29]	Tumor regression with regional distribution of the targeted toxin TF-CRM107	Transferrin receptor/Tf-CRM107	First major human proof-of-concept that regional receptor-targeted toxin delivery could produce objective responses in malignant brain tumors.
1999	Debinski et al. [30]	Receptor for interleukin 13 is a marker and therapeutic target for human high-grade gliomas	IL13Rα2 target validation	Established IL13R as a therapeutic target in human gliomas and provided the biologic basis for later IL13-toxin programs.
2000	Rand et al. [31]	Intratumoral administration of recombinant circularly permuted interleukin-4-Pseudomonas exotoxin in patients with high-grade glioma	IL-4 receptor/cpIL4-PE	First-in-human IL-4 toxin study showing feasibility, tumor necrosis, and limited systemic toxicity in glioma.
2000	Debinski et al. [30]	Expression of a restrictive receptor for interleukin 13 is associated with greater malignancy of human gliomas	IL13Rα2 expression/target validation	Clarified the restricted IL13-binding phenotype in gliomas and strengthened the rationale for IL13R-targeted therapy.
2002	Kunwar et al. [32]	Convection enhanced delivery of IL13-PE38QQR for treatment of recurrent malignant glioma	IL13Rα2/IL13-PE38QQR via CED	One of the earliest human CED toxin trials in glioma, establishing catheter-based locoregional toxin infusion as a practical translational platform.
2002	Vallera et al. [33]	Targeting Urokinase-Type Plasminogen Activator Receptor on Human Glioblastoma Tumors With Diphtheria Toxin Fusion Protein DTAT	uPAR/DTAT	Seminal uPAR-targeting paper that validated uPAR as a glioma toxin target and influenced later bispecific toxin design.
2003	Weaver et al. [34]	Transferrin receptor ligand-targeted toxin conjugate (Tf-CRM107) for therapy of malignant gliomas	Transferrin receptor/Tf-CRM107	Consolidated the transferrin receptor toxin program and summarized the first human translational experience with Tf-CRM107.
2003	Husain et al. [35]	Interleukin-13 receptor-directed cytotoxin for malignant glioma therapy	IL13Rα2/IL13-PE38QQR	Key preclinical validation of IL13Rα2 as a glioma target, directly enabling the later IL13 toxin clinical program.
2007	Kunwar et al. [36]	A Report by the Cintredekin Besudotox Intraparenchymal Study Group	IL13Rα2/Cintredekin besudotox	Consolidated the IL13-PE38QQR clinical program and highlighted delivery quality as a major determinant of outcome.
2008	Sampson et al. [37]	Intracerebral infusion of an EGFR-targeted toxin in recurrent malignant brain tumors	EGFR/TP-38	Landmark human EGFR-targeted toxin report that broadened receptor-toxin therapy beyond IL13Rα2 and TfR.
2009	Mardor et al. [38]	Convection-enhanced drug delivery of interleukin-4 Pseudomonas exotoxin (PRX321): increased distribution and magnetic resonance monitoring	IL-4 receptor/PRX321	Important delivery-optimization paper showing that imaging and CED control could improve the dispersion of IL-4 toxin in glioma.
2010	Kunwar et al. [39]	Phase III randomized trial of CED of IL13-PE38QQR vs. Gliadel wafers	IL13Rα2/IL13-PE38QQR vs. Gliadel	Pivotal randomized CED trial in recurrent GBM; feasible delivery was shown, but efficacy depended heavily on distribution and trial design.
2010	Candolfi et al. [40]	Gene therapy-mediated delivery of targeted cytotoxins for glioma therapeutics	Targeted cytotoxin gene delivery	Useful bridge paper showing how local gene delivery could be used to sustain targeted toxin activity in glioma.
2017	Rossmeisl et al. [41]	P08.12 Tolerability and initial efficacy of convection-enhanced delivery of combinatorial IL-13RA2 and EphA2 targeted cytotoxins to dogs with spontaneous intracranial malignant gliomas	IL13Rα2 + EphA2/combinatorial cytotoxins	Important translational large-animal CED study that extended dual-target toxin delivery beyond rodent models.
2019	Heiss et al. [42]	Phase I trial of convection-enhanced delivery of IL13-Pseudomonas toxin in children with diffuse intrinsic pontine glioma	IL13Rα2/IL13-Pseudomonas toxin	Extended IL13-toxin/CED into pediatric DIPG and showed feasibility in a highly challenging clinical setting.
2023	Sampson et al. [43]	Targeting the IL4 receptor with MDNA55 in patients with recurrent glioblastoma	IL-4 receptor/MDNA55 (bizaxofusp)	Modern IL-4R toxin clinical milestone showing the platform remains active in current glioma translation.

**Table 2:** IL-4 toxin-based therapies in human glioma studies through 2025. Published human studies of IL-4 receptor-targeted toxin therapies in glioma, summarizing the clinical development of cpIL4-PE, NBI-3001/IL4-PE, and MDNA55 (bizaxofusp).

Year	Author	Study	PMID	Target	Population	Main Finding
2000	Rand et al. [31]	Intratumoral administration of recombinant circularly permuted interleukin-4-Pseudomonas exotoxin in patients with high-grade glioma	10873064	IL4R/IL-4(38-37)-PE38KDEL (cpIL4-PE)	Recurrent malignant high-grade gliomas	Direct intratumoral infusion was safe without systemic toxicity; 6 of 9 patients showed tumor necrosis; one patient achieved complete remission lasting >18 months.
2001	Kawakami et al. [84]	Overexpressed cell surface interleukin-4 receptor molecules can be exploited to target toxins to human glioblastoma multiforme	11642612	IL4R/IL-4(38-37)-PE38KDEL (cpIL4-PE)	Recurrent glioblastoma multiforme, phase I	Preliminary clinical results showed antitumor activity against recurrent GBM without detectable systemic toxicity, supporting IL-4R as a therapeutic target.
2002	Weber et al. [85]	Safety, tolerability, and tumor response of IL4-Pseudomonas exotoxin (NBI-3001) in patients with recurrent malignant glioma	12952293	IL4R/NBI-3001 (IL4-PE)	Recurrent malignant glioma (GBM and AA)	Intratumoral NBI-3001 showed an acceptable safety profile; median OS was 8.2 months overall, 5.8 months for GBM; MRI showed tumor necrosis in many patients.
2023	Sampson et al. [43]	Targeting the IL4 receptor with MDNA55 in patients with recurrent glioblastoma	36640127	IL4R/MDNA55 (bizaxofusp)	Recurrent GBM	Single-treatment MDNA55 via CED showed tumor control and promising survival; median OS 11.64 months (ITT), 12-month OS 43%; primary endpoint met.

**Table 3:** IL-13-based toxin therapies in human glioma studies through 2025.

Year	Author	Study	PMID	Target	Population	Main Finding
2002	Kunwar et al. [32]	CED of IL13-PE38QQR for the treatment of recurrent malignant glioma	14531568	IL13Rα2/IL13-PE38QQR	Recurrent malignant glioma	Early human CED experience showing the feasibility of locoregional delivery and initial safety/distribution data.
2007	Kunwar et al. [36]	A Report by the Cintredekin Besudotox Intraparenchymal Study Group	17327604	IL13-PE38QQR	Recurrent malignant glioma	Summarized the linked intraparenchymal studies and helped formalize the translational path for IL13-PE38QQR.
2010	Kunwar et al. [39]	Phase III randomized trial of CED of IL13-PE38QQR vs. Gliadel wafers	20511192	IL13-PE38QQR	Recurrent GBM	Showed that the therapy could be delivered, but it did not outperform Gliadel on survival endpoints.
2019	Heiss et al. [42]	Phase I trial of convection-enhanced delivery of IL13-Pseudomonas toxin in children with diffuse intrinsic pontine glioma	30544335	IL13-PE38QQR	DIPG	Phase I CED study in 5 children showed feasibility, transient neurologic toxicities that resolved, and short-term radiographic antitumor effects in 2 of 5 patients.
2021	Rossmeisl et al. [103]	Phase I trial of CED of IL13RA2 and EPHA2 immunotoxins	32812637	IL13Rα2- and EphA2-directed immunotoxins	Recurrent malignant glioma	Demonstrated modern dual-target CED translation building on the earlier IL-13 toxin experience.
2022	Rechberger et al. [104]	Locoregional infusion of IL13Rα2-specific immunotoxins in children	35872639	IL13Rα2-specific immunotoxins	DIPG/HGG/DMG	Confirmed pediatric locoregional feasibility and safety, extending the IL-13 toxin lineage into childhood glioma.

**Table 4:** EGFR toxin-based therapies in human glioma studies through 2025. Published human studies of EGFR-targeted toxin therapy in glioma, focused on the TP-38 clinical program and its intracerebral delivery studies.

Year	Author	Study	PMID	Target	Population	Main Finding
2003	Sampson et al. [122]	Progress report of a Phase I study of the intracerebral microinfusion of a recombinant chimeric protein composed of transforming growth factor-alpha and a mutated form of Pseudomonas exotoxin termed PE-38 (TP-38) for the treatment of malignant brain tumors	14649883	EGFR/TP-38 (TGF-α–PE38)	Recurrent malignant brain tumors	Demonstrated feasibility of intracerebral delivery, with radiographic responses including one durable complete response.
2004	Sampson et al. [123]	Sustained radiographic and clinical response in patient with bifrontal glioblastoma following intracerebral infusion of TP-38	15701286	EGFR/TP-38	Recurrent bifrontal glioblastoma	Single-patient report showing prolonged radiographic and clinical benefit after TP-38.
2008	Sampson et al. [37]	Intracerebral infusion of an EGFR-targeted toxin in recurrent malignant brain tumors	18403491	EGFR/TP-38	Recurrent malignant brain tumors	Full clinical report extending the TP-38 experience and supporting safety/activity of EGFR-targeted toxin delivery.

**Table 5:** Transferrin receptor toxin-based therapies in human glioma studies through 2025. Published human studies of transferrin receptor-targeted toxin therapy in glioma, centered on the Tf-CRM107 clinical experience.

Year	Author	Study	PMID	Target	Population	Main Finding
1997	Laske et al. [29]	Tumor regression with regional distribution of the targeted toxin TF-CRM107	9396606	Transferrin receptor/Tf-CRM107	Recurrent malignant brain tumors	Regional perfusion via interstitial microinfusion produced tumor responses without symptomatic systemic toxicity; 9/15 evaluable patients had ≥50% volume reduction and 2 complete responses.
2003	Weaver et al. [34]	Transferrin receptor ligand-targeted toxin conjugate (Tf-CRM107) for therapy of malignant gliomas	14649881	Transferrin receptor/Tf-CRM107	Review of phase I/II human malignant glioma studies	Summarized the phase I/II human experience, including 35% complete/partial response among evaluable patients in the phase II program, and concluded that the data justified further development.

## Data Availability

No new data were created or analyzed in this study.
